# Medically tailored meals for the management of symptomatic ascites: the SALTYFOOD pilot randomized clinical trial

**DOI:** 10.1093/gastro/goaa059

**Published:** 2020-11-12

**Authors:** Elliot B Tapper, Jad Baki, Samantha Nikirk, Scott Hummel, Sumeet K Asrani, Anna S Lok

**Affiliations:** 1 Division of Gastroenterology and Hepatology, University of Michigan, Ann Arbor, MI, USA; 2 Gastroenterology Section, VA Ann Arbor Healthcare System, Ann Arbor, MI, USA; 3 Department of Biostatistics, University of Michigan, Ann Arbor, MI, USA; 4 Baylor University Medical Center, Dallas, TX, USA

**Keywords:** cirrhosis, ascites, paracentesis, sodium

## Abstract

**Background:**

Ascites is a costly, morbid complication of cirrhosis. Although a low-sodium diet is central to the clinical management of ascites, its efficacy is limited by poor adherence. We aimed to determine the feasibility and impact of low-sodium medically tailored meals (MTM) intervention.

**Methods:**

We enrolled 40 persons with cirrhosis and ascites at the time of a paracentesis in a 12-week, 1:1 randomized trial of standard of care (SOC) (low-sodium diet educational handout) or MTM with <2,000 mg of sodium, >2,100 kcal, and >80 g of protein including a nocturnal protein supplement. We determined the proportion of eligible candidates recruited and adherence to MTM. The primary outcome was the number of paracenteses performed during weeks 0–12. We also collected ascites-specific quality-of-life (ASI-7) scores.

**Results:**

The median age of the enrolled subjects was 54 (IQR, 47–63) years, 46% were female, with median MELD-Na 18 (IQR, 11–23) and albumin 2.7 (IQR, 2.5–3.3) g/dL. At baseline, subjects reported a median of two (IQR, 1–3) paracenteses in the prior 4 weeks. Adherence to the meal schedule was excellent save for when hospitalizations occurred. After 12 weeks, patients in the MTM arm required fewer paracenteses per week than those in the SOC group [median (IQR): 0.34 (0.14–0.54) vs 0.45 (0.25–0.64)]. During the trial, four (20%) SOC patients died, whereas two (10%) died and one (5%) was transplanted in the MTM arm. Ascites-specific quality of life improved to a greater degree in the MTM arm compared to the SOC arm, by 25% (IQR, –11% to 61%) vs 13% (IQR, –28% to 54%).

**Conclusion:**

A trial of MTM for persons with ascites is feasible and potentially effective. Both arms experienced benefits, highlighting the role for improved education and closer monitoring in this challenging condition.

## Introduction

Ascites—the most common complication of cirrhosis, occurring in as many as 50% of patients—is associated with life-threatening infections, renal dysfunction, malnutrition, diminished health-related quality of life (HRQOL), and a <50% 2-year survival [1]. The personal and population burden of ascites drives a significant portion of morbidity, mortality, and healthcare costs of cirrhosis [2]. However, the effective management of ascites can be very challenging.

First-line therapy for ascites is dietary-salt restriction (2,000 mg/88 mmol per day) [3]. Dietary therapy alone, however, will only result in ascites control in 14% of patients [4]. Complicating matters further, sodium restriction may complicate global nutritional goals. Patients who successfully restrict sodium after nutritionist consultation also, inadvertently, reduce caloric intake by >17% [5]. Diuretic therapy is a useful adjunct for the management of ascites but it is also associated with significant side effects (e.g. renal injury, hyper/hypokalemia, hyponatremia). For patients with refractory ascites and those intolerant to diuretics, regular therapeutic paracentesis is the only resort unless the patient is a candidate for transjugular intra-hepatic portosystemic shunt or liver transplantation. Although nutritional interventions have been examined in trials, such studies are dated, of variable quality, and often do not utilize current nutritional recommendations [6].

We hypothesized that the efficacy of a low-sodium diet could be improved by teaching patients how to prepare palatable low-sodium meals. Medically tailored meals (MTM) have been used to reduce readmissions for congestive heart failure and improve healthcare utilization among food-insecure community-dwelling elders [7, 8]. Short-term MTM interventions are increasingly supported by payors and can be leveraged using existing resources such as meals-on-wheels [8]. Patients with cirrhosis and ascites are natural candidates for MTM interventions. We designed a trial (SALTYFOOD) to determine the feasibility and impact of MTM on clinical and patient-reported outcomes in patients with cirrhosis and severe ascites.

## Patients and methods

We conducted a 12-week pilot, single-centre, randomized, controlled trial of MTM in 40 persons with cirrhosis and severe/symptomatic ascites. All patients were required to have a history of multiple paracenteses including at least one within the prior 30 days. We excluded patients with creatinine >1.5 mg/dL, planned liver transplantation, or portosystemic-shunt procedure. All patients were enrolled at the time of their outpatient paracentesis or hospitalization for symptomatic ascites by the principle investigator and/or trained clinical-research co-ordinator. This study was approved by the Michigan Medicine institutional review board (HUM00141457). All patients provided written consent prior to participation.

All patients were randomized 1:1 to receive standard of care (SOC, an educational handout on low-sodium diets) or MTM with <2,000 mg of sodium, >2,100 kcal, and >80 g of protein, including a nocturnal protein supplement in addition to SOC (trial registration NCT03493204) [9]. The food intervention took place from weeks 0–4 and consisted of three meals each day selected by the patient from a menu and delivered to their home on a weekly basis. The menu was designed by registered dieticians and the food was prepared by a central kitchen (Mom’s Meals Nourishcare, a USDA-approved delivery service). The nocturnal protein supplement (ProCel Chocolate, 15 g whey protein) could be dissolved in any liquid and was provided to patients in a canister at the time of enrolment. All patients were followed for 12 weeks with phone assessments of diet and health state at weeks 1, 2, 4, 6, and 8.

All patients had an in-person 12-week visit. All follow-up assessments were performed by research coordinators. Feasibility outcomes included the proportion of (i) eligible candidates who were randomized, (ii) food recipients who continued the intervention without crossover, and (iii) adherence to diet. The primary outcome was the number of paracenteses per person-week performed during weeks 0–12 analysed in an intention-to-treat fashion. In-person baseline and end-of-study assessments included measures of liver function [model for end-stage liver disease (MELD)-Na, albumin], frailty/disability [handgrip measured in pounds by dynamometer, Katz activity of daily living (ADL), and falls], diuretic dose (separately evaluating loop diuretics—as furosemide-equivalent milligrams—and aldosterone antagonists), and ascites-specific quality of life [ascites symptom invertory-7 (ASI-7) scores, range 0–28 [10]; higher scores indicating worse ascites control]. Patients were followed until end-of-study, death, or transplantation. Adherence to the diet was assessed using a daily diet diary (number of meals consumed and additional foods eaten). All outcomes were assessed as intention-to-treat.

Statistical analyses were deferred in the setting of this pilot trial for lack of adequate power to detect differences in clinical outcomes. We determined a 40-subject sample size in order to evaluate the time required for one site to recruit a moderate number of patients and allow sufficient variability of experience that no one patient would bias results. The randomization procedure allowed replacement if a patient was enrolled but never were initiated into the study.

## Results

### Feasibility

Seventy-eight potentially eligible patients were screened between June 2018 and June 2019; 35 declined participation and 43 (55%) were enrolled. The primary reason for declining participation was refusal to participate in a meals intervention owing to preference for own cooking or food sensitivities. Three patients were disenrolled before initiating the trial: one for death on the day of hospital discharge and two for admission to a long-term nursing facility. Of the 40 patients included in the trial analysis, two patients discontinued meal delivery within 2 weeks, one for Crohn’s flare and the other for severe nausea. These patients crossed over to the control arm to complete the trial (but were analysed as part of the MTM arm given the intention-to-treat design). Patients reported that the intervention was well received, though patients with poor dentition or lactose intolerance suggested more tailored meal choices. Beyond these two, the other patients reported complete adherence to the meal schedule save for when hospitalizations occurred.

### Baseline characteristics

At enrolment, the median age was 54 [interquartile range (IQR), 47–63] years, 73% had <$75,000 household income, 55% had <high school education, 46% were female, 44% diabetic, 44% fully ADL-independent, with median MELD-Na 18 (IQR, 11–23), median creatinine 0.88 (IQR, 0.69–1.16) mg/dL, and median albumin 2.7 (IQR 2.5–3.3) g/dL. At baseline, subjects reported a median of two (range, one to three) therapeutic paracenteses in the prior 4 weeks. The median visual analogue scale (VAS) was 50 (IQR 40–70) and ASI-7 was 18 (range 11–21). The median handgrip was 25 (IQR, 18–33) pounds. Median baseline loop diuretics and aldosterone-antagonist doses were 40 (IQR, 25–80) mg and 100 (IQR, 50–100) mg, respectively.

### Clinical outcomes

**Figure 1.**
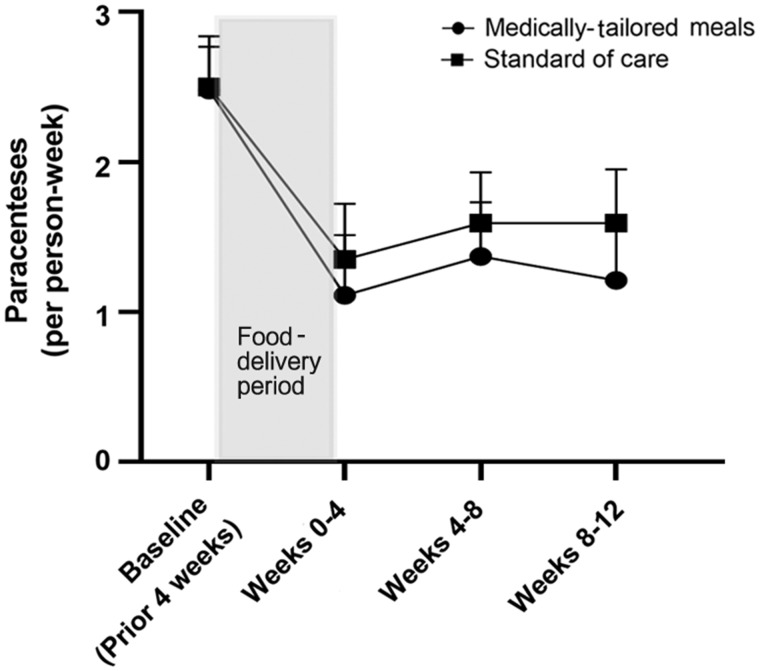
Paracenteses performed per cirrhosis patient with severe ascites over time in a trial of medically tailored meals

By week 12, four patients (20%) in the SOC arm had died and none had been transplanted, whereas two (10%) in the MTM arm had died and one (5%) had undergone liver transplantation. Patients in the MTM arm required fewer paracenteses than those in the SOC arm over the entire study period [median (IQR): 0.34 (0.14–0.54) vs 0.46 (0.25–0.64) per week]. The number of paracenteses required over time is depicted in Figure 1. Ascites-specific quality of life was improved to a greater degree in the MTM arm compared to the SOC arm, by 25% (IQR, –11% to 61%) vs 13% (IQR, –28% to 54%). Frailty measures were unchanged. Compared to patients in the SOC arm, those receiving MTM spent fewer days in the hospital (0.62 ± 1.20 vs 1.04 ± 1.19 days/person-week). Outcomes are summarized in Table 1. Compared to the MTM arm, loop diuretic dosing in the SOC arm increased by 31.4% and aldosterone-antagonist doses increased by 167%.

**Table 1.** Clinical outcomes of patients with cirrhosis and severe ascites

Outcome	Medically tailored meals (*n* = 20)	Standard of care (*n* = 20)
Number of paracentesis (per person)		
Week 0–1	0 (0–0)	0 (0–1)
Week 1–2	0 (0–1)	0 (0–1)
Week 2–4	0 (0–1)	0.5 (0–1)
Week 4–8	1 (0–3)	1 (0.25–2)
Week 8–12	1 (0–2)	2 (0–2.75)
Per person-week	0.34 (0.14–0.54)	0.45 (0.25–0.64)
Change in VAS (higher better)	43% (–12% to 99%)	33% (0% to 66%)
Change in ASI-7 (lower better)	–25% (–61% to 11%)	–13% (–54% to 28%)
Change in handgrip (higher better)	0% (–22% to 22%)	–9% (–30% to 11%)
Mortality	2 (10%)	4 (20%)
Transplantation	1 (5%)	0 (0%)
Hospital stay per person-week, days	0.62 ± 1.20	1.04 ± 1.19
Change in loop diuretics (furosemide-equivalent doses)	11% (–27% to 49%)	20% (–32% to 73%)
Change in aldosterone antagonists	14% (–35% to 62%)	30% (–52% to 112%)

Data are presented as median (interquartile range), mean ± standard deviation, and *n* (%).

Changes compared week 12 end-of-trial values to baseline.

VAS, visual analogue scale; ASI-7, ascites symptom inventory-7.

## Discussion

This pilot, randomized trial of MTM with low-sodium/high-protein content demonstrates the feasibility of the intervention. Further, these data show that MTM has the potential to reduce the need for therapeutic paracentesis and improving the quality of life in patients with cirrhosis and symptomatic ascites. We also found that both the control and MTM arms experienced reduced paracentesis frequency and improved ascites-specific HRQOL benefits. These data highlight the benefits of standardized education and closer monitoring in the management of ascites.

This pilot trial demonstrated the feasibility and acceptance of home-delivered MTM and yielded promising results. However, a future trial aimed at improving outcomes must incorporate multiple changes. First, an increased sample size will be needed to detect differences in clinical outcomes. A multicentre trial in two different regions of the USA is planned. Second, given the high competing risk of death or transplantation for patients with ascites, future trials should expand the inclusion criteria to include patients with moderate ascites. We focused our enrolment on persons with a high symptom burden as a function of enrolment at the time of a paracentesis. As a consequence, we enrolled a sick population in whom the severity of portal hypertension could limit the efficacy of a nutritional intervention. A trial that enrols patients with a lesser ascites burden could then focus on reducing the progression of disease. For this reason, we will extend the next study period to 52 weeks, increase the MTM-intervention period to 8 weeks, and enrol patients with a lower ascites burden (limiting to one paracentesis in the prior month). Third, future trials must use rigorous methods to assess adherence to the delivered meals and food intake outside of the delivered meals (in both the intervention and control arms). In our upcoming trial, we will be using unscheduled 24-hour dietary recalls performed by trained dieticians as well as urinary sodium-excretion metrics. The use of dietary-recall methods will also allow us to assess whether the trial was associated with a durable change in diet quality following on after the intervention period, i.e. did the examples of low-sodium high-protein meals provide guidance for subsequent home-prepared meals? Finally, the cost-effectiveness of this intervention is central to its scalability. While the cost of the meals can be low (∼$150–200 per week), this would be substantial at the population level. The future trial will incorporate a formal costing analysis to account for trade-offs (cost of paracentesis, hospital-days), cost-effectiveness (relative changes in utility vis-a-vis HRQOL and survival), and personalization (using predictive modelling to select patients most likely to benefit).

In conclusion, MTM is a promising intervention for patients with cirrhosis. Based on these data, we are planning a larger-scale, multicentre, randomized trial of patients with cirrhosis and moderate-to-severe ascites with clinical and patient-reported outcomes as well as the cost-effectiveness of MTM.

## Author’s contributions

E.T. is the guarantor of this article. Concept: E.T. and S.H.; Data acquisition: E.T., J.B., and S.N.; Data analysis: E.T. and A.S.L.; Manuscript drafting: E.T.; Critical revision: S.H., S.N., J.B., S.K.A., and A.S.L.

## Funding

E.T. receives funding from the National Institutes of Health through the Michigan Institute for Clinical and Health Research [KL2TR002241] and NIDDK [1K23DK117055-01A1].
